# Revisiting Papillomavirus Taxonomy: A Proposal for Updating the Current Classification in Line with Evolutionary Evidence

**DOI:** 10.3390/v14102308

**Published:** 2022-10-21

**Authors:** Koenraad Van Doorslaer

**Affiliations:** 1 Genetics Graduate Interdisciplinary Program, University of Arizona, Tucson, AZ 85719, USA; vandoorslaer@arizona.edu; 2 School of Animal and Comparative Biomedical Sciences, University of Arizona, Tucson, AZ 85721, USA; 3 The BIO5 Institute, Department of Immunobiology, Cancer Biology Graduate Interdisciplinary Program, UA Cancer Center, University of Arizona Tucson, Tucson, AZ 85724, USA

**Keywords:** *Papillomaviridae*, evolution, taxonomy

## Abstract

Papillomaviruses infect a wide array of animal hosts and are responsible for roughly 5% of all human cancers. Comparative genomics between different virus types belonging to specific taxonomic groupings (e.g., species, and genera) has the potential to illuminate physiological differences between viruses with different biological outcomes. Likewise, extrapolation of features between related viruses can be very powerful but requires a solid foundation supporting the evolutionary relationships between viruses. The current papillomavirus classification system is based on pairwise sequence identity. However, with the advent of metagenomics as facilitated by high-throughput sequencing and molecular tools of enriching circular DNA molecules using rolling circle amplification, there has been a dramatic increase in the described diversity of this viral family. Not surprisingly, this resulted in a dramatic increase in absolute number of viral types (i.e., sequences sharing <90% L1 gene pairwise identity). Many of these novel viruses are the sole member of a novel species within a novel genus (i.e., singletons), highlighting that we have only scratched the surface of papillomavirus diversity. I will discuss how this increase in observed sequence diversity complicates papillomavirus classification. I will propose a potential solution to these issues by explicitly basing the species and genera classification on the evolutionary history of these viruses based on the core viral proteins (E1, E2, and L1) of papillomaviruses. This strategy means that it is possible that a virus identified as the closest neighbor based on the E1, E2, L1 phylogenetic tree, is not the closest neighbor based on L1 nucleotide identity. In this case, I propose that a virus would be considered a novel type if it shares less than 90% identity with its closest neighbors in the E1, E2, L1 phylogenetic tree.

## 1. Intro to Papillomavirus Biology

Members of the *Papillomaviridae* family primarily infect mucosal and keratinized epithelia. While the exact evolutionary history of papillomaviruses is complex, these viruses have evolved alongside their host for 400–450 million years [1,2,3,4]. As a result, most infections are asymptomatic, but a subset of the papillomaviruses has been associated with specific lesions and cancers [5]. All (known) papillomaviruses encode a core set of viral proteins [3]. The early (E1 and E2) proteins play key roles in regulating viral transcription and replication [6,7]. The late (L1 and L2) genes encode the viral structural proteins [8,9]. These 4 core proteins (E1, E2, L1, and L2) can be identified in all papillomaviruses sequenced to date [10,11]. The viral helicase, E1, is essential for the replication and amplification of the viral chromosome in the nucleus of infected cells [6]. The E2 protein regulates viral transcription, initiation of DNA replication, and partitioning of the viral genome [7]. The additional viral proteins likely play essential yet supporting roles in the viral lifecycle. The E6 and E7 proteins are critical in creating a cellular milieu that supports the viral lifecycle [12,13] by uncoupling viral replication from cellular differentiation. In a subset of papillomaviruses, expression of E6 and E7 is associated with cancer progression [14].

Expression of viral mRNA is temporally synced with tissue differentiation and is tightly regulated at the level of transcription and RNA processing [15,16,17]. In addition to these viral proteins, most papillomavirus types also express an E1^E4 and E8^E2 gene product [18,19]. Finally, a subset of viral mRNA encodes a short, hydrophobic, transmembrane protein, E5 or E10. E5 proteins are typically encoded in the 3′-end of the early coding region [20,21]. The E10 proteins are located in this region without an E6 gene [11].

## 2. Current Classification of Viruses in the Family *Papillomaviridae*

The seventh International Committee on Taxonomy of Viruses (ICTV) report established the family *Papillomaviridae* as a taxonomic unit [22]. The family *Papillomaviridae* consists of a diverse group of viruses with a circular double-stranded DNA genome ranging between 5–8.5 kb in size. Genetically distinct papillomavirus types have been described in fish, reptiles, and many mammals [1,2].

**Figure 1:**
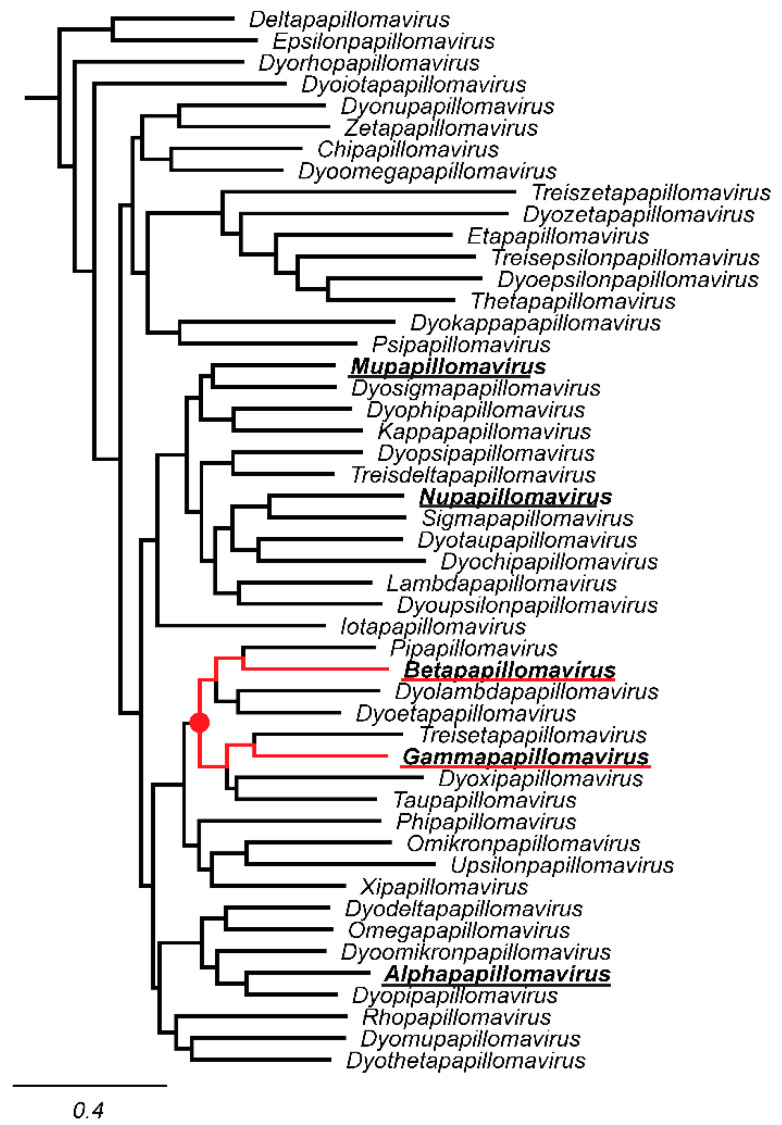
Current genera within the *Firstpapillomavirinae* sub-family. A maximum likelihood phylogenetic tree based on L1 nucelotide sequence was constructed using FastTree [27]. Individual genera were represented by a single papillomavirus type and are shown. The sequences in the phylogenetic tree can be classified into 49 genera. Genera that contain human papillomavirus types are bolded and underlined. The most recent common ancestor between the *Betapapillomavirus* and *Gammapapillomavirus* genera (underlined in red) is indicated by a red filled in circle.

The family *Papillomaviridae* are classified into the order *Zurhausenvirales*, class *Papovaviricets*, phylum *Cossaviricota*, kingdom *Shotokuvirae* and realm *Monodnaviria* [23]. Within the family *Papillomaviridae*, classification has traditionally been based on nucleotide sequence identity [24]. Specifically, nucleotide pairwise identity of the viral L1 open reading frame (ORF) serves as the basis for this classification [24,25]. As formalized in a landmark paper, authored by the members of the ICTV Papillomaviruses study group, papillomaviruses were assigned to genera named using the Greek alphabet in the prefix of the word papillomavirus [24]. Within genera, specific viral “types” are assigned to species and the species named using the genus names with a number suffix, thus fulfilling the binomial naming convention [26] (Figure 1).

The current approach requires the L1 sequence of distinct viral types to be aligned, and pairwise sequence identities are used to define the demarcation between types, species, and genera. In this approach, L1 sequences with pairwise identities of (1) >90% belong to the same type; (2) >70% belong to the same species; (3) >60% to the same genera. 

For example, human papillomavirus 16 is a type in the species *Alphapapillomavirus 9*, the genus *Alphapapillomavirus*, and the sub-family *firstpapillomavirinae*. This nomenclature system was based on the data available at that time and was universally accepted by the papillomavirus community. 

## 3. Importance of Papillomavirus Classification for Comparative Genomics

Comparative genomics uses a variety of tools to compare the complete genome sequences of different viruses. This approach allows researchers to pinpoint physiologically relevant similarities and differences between different papillomaviruses. By analyzing the evolutionary relationships between viral genomes and the corresponding differences in their DNA (and their genes) we can understand how these genes impact the viral lifecycle and oncogenic progression. In turn, this may translate into innovative approaches for diagnosing, preventing, or treating human disease and thereby improving human health. For example, an evolutionary relationship with HPV16 is used to extrapolate clinical risk for oncogenic progression [28,29,30,31].

However, comparative genomics requires a robust (taxonomic) classification system that is based on the evolutionary history of the viruses while, ideally, reflecting physiological similarities and differences. The establishment of the papillomavirus episteme (PaVE; [1,2]) has been hugely advantageous in normalizing the study of papillomavirus genome diversity. However, I believe that it is time to update the viral classification to reflect the current viral diversity. Importantly, these views are mine and may not reflect the current position of the ICTV papillomavirus study group.

## 4. Dramatic Increase in the Number of Viral Types, Species, and Genera

**Figure 2:**
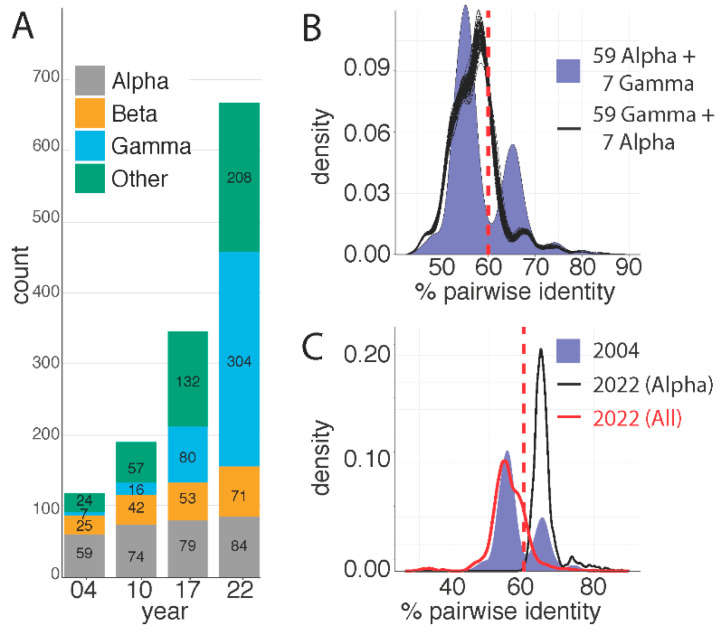
Papillomavirus L1 sequences pairwise nucleotide comparison is skewed by number of viral types. (**A**) Number of viral types classified as belonging to the *Alphapapillomavirus*, *Betapapillomavirus*, *Gammapapillomavirus*, or other genera are indicated over time. 2004 corresponds to the paper by de Villiers and colleagues [24]; 2010 is based on the updated classification as published by Bernard and colleagues [32]. The 2017 and 2022 timepoints are based on data in the papillomavirus episteme [1,2]. (**B**) The purple plot reproduces the results of the initial classification proposal [24] based on 59 *Alphapapillomavirus* types and 7 *Gammapapillomavirus* types. The valley at ~60% (red dotted line) represents the current genus demarcation. *Gammapapillomavirus* (*n* = 59) and *Alphapapillomavirus* (*n* = 7) were randomly selected from the currently known diversity on the Papillomavirus episteme. The remaining 49 viruses were kept identical to the types used in 2004. Pairwise identities were calculated and plotted. This was repeated 100 times (black plots). (**C**) Plots of subsets of pairwise sequence alignments. The purple plot reproduces the results of the initial classification proposal [24], red plot corresponds to the currently known diversity, while the black curve shows the distribution of pairwise comparisons of types belonging to the genus *Alphapapillomavirus*.

The seminal paper describing the classification of papillomaviruses [24], used 118 unique, eligible papillomaviruses to define demarcation criteria. Based on pairwise sequence identity of the L1 ORF, these 118 viruses were classified into 14 genera and 43 species. Fifty-nine virus types (50%) representing 15 species were assigned to the genus *Alphapapillomavirus* and 29 virus types representing 5 species were assigned to *Betapapillomavirus*. At the time of the initial classification, only seven virus types (5 species) were assigned to the genus *Gammapapillomavirus* and the remaining 27 virus types were classified in 19 species and assigned to 13 genera (Figure 1). The increase in the number of papillomavirus types is reflected in an even more dramatic increase in the number of species and genera (Figure 2A; based on sequences used for previous classification [24,32] or the PaVE database on 8/17/2017 (*n* = 340), and 7/3/2022 (*n* = 667), respectively). Indeed, many of the newly identified viruses are the sole member of a novel species within a novel genus (i.e., singletons), highlighting that we have only scratched the surface of papillomavirus diversity. Practically, this limits the value of viral species or genera to inform comparative genomics experiments. 

Furthermore, since the initial classification, the number of virus types classified in the genus *Alphapapillomavirus* has marginally increased, while the number of types in the genus *Gammapapillomavirus* has increased dramatically (Figure 2).

## 5. L1 Based, Pairwise Identity Distribution Is Unique to the *Alphapapillomavirus* Genus

Based on papillomavirus sequence data available in 2004, a histogram of the distribution of L1 open reading frame pairwise sequence identities show a clear multimodal distribution (filled blue plot in Figure 2B). This graph recapitulates the data in the paper by de Villiers and colleagues [24], the valley at approximately 60% identity is the basis for the current genera demarcation. However, since 2004, there has been an increase in the identification of new papillomaviruses providing a better understanding of their diversity (Figure 2A). This implies that, while the papillomavirus types used in the 2004 analysis for the purpose of classification represented the known viral diversity at the time, the original ‘training’-set used to determine relationships between papillomavirus types is no longer representative of the known diversity today. In 2004, 118 viruses were classified, including 59 viruses in the genus *Alphapapilloamvirus* and only 7 viruses in the genus *Gammapapillomavirus*. To test how the largest group of viruses would affect the classification criteria, I plotted the pairwise sequence identities of a simulated set of sequences (Figure 2B). As in 2004, 118 viruses were used, however, 59 virus types classified in the genus *Gammapapillomavirus* were randomly chosen from the diversity known today. At the same time, only seven (random) types classified in the genus *Alphapapillomavirus* were included. Hence, this replicates the analysis performed in 2004, but the number of types within the *Alphapapillomavirus and Gammapapillomavirus* genera were flipped. I repeated this analysis 100 separate times and plotted the results of each simulation (black lines in Figure 2B). Unlike what we see in the blue histogram, the valley around 60% is not reproduced in any of these simulations. This suggests that the design of the papillomavirus classification was biased by the number of sequences in the genus *Alphapapillomavirus*. Figure 2C shows pairwise sequence comparisons between different samples of the data. The purple histogram again shows the 118 viruses used in 2004, while the red curve compares all viruses known (i.e., in the PaVE database) today. The black curve shows the pairwise identity between all viruses classified to the genus *Alphapapillomavirus*. It is clear that the current known papillomavirus diversity (red curve) does not recapitulate the valley around 60%. Furthermore, it appears that 60% cutoff is driven by pairwise identities between members of the genus *Alphapapillomavirus*. Indeed, the peak of the black curve overlaps with the second peak of the multimodal blue curve. In conclusion, increased sampling has changed the distribution from multimodal to a (skewed) unimodal distribution (red line in Figure 2C).

## 6. Genetic Saturation within the L1 Gene

**Figure 3:**
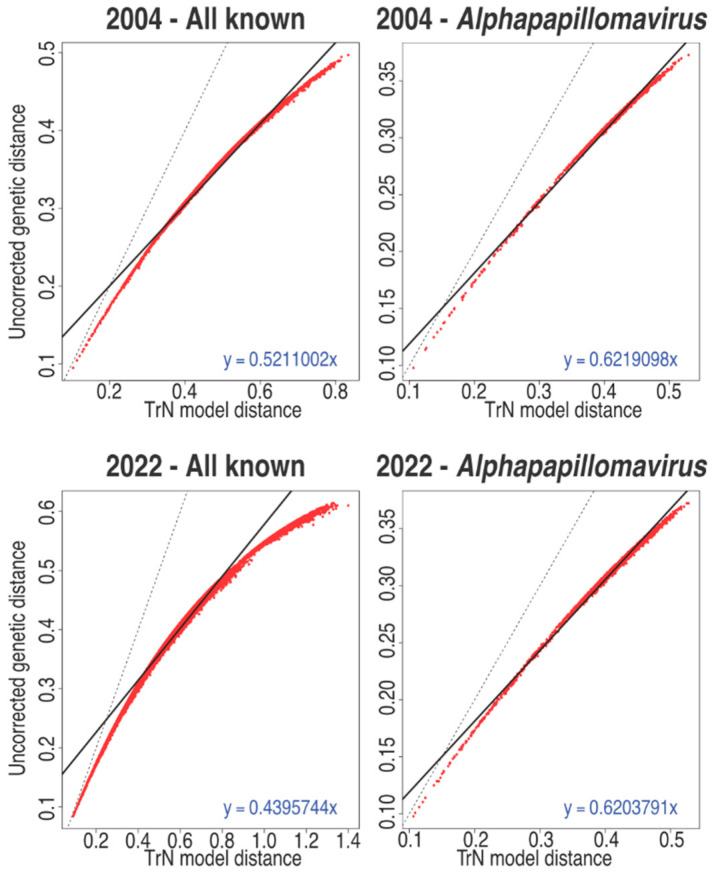
L1 genetic saturation plots. The L1 DNA sequence of all sequences belonging to a specific subset of the data were aligned at the amino acid level using MAFFT [35,36]. The protein alignments were back translated to nucleotides. The uncorrected distance is plotted versus the model corrected distance (red dots). The solid black line represents the best linear fit of the data (equation given) while the dotted black line represents a perfect match between the corrected and uncorrected distances.

Nucleotide sequence alignments are optimal to infer diversity between closely related viruses. However, nucleotide sequence alignments are sensitive to genetic saturation. Genetic saturation can be caused by multiple substitutions at the same site in a sequence, or identical nucleotide changes in a different sequence. When comparing sequences, genetic saturation makes the apparent sequence divergence rate lower than the occurred divergence between two sequences. Genetic saturation complicates the interpretation of the percentage of nucleotide divergence between two sequences [33] and could therefore falsely group diverse sequences into the same species or genus. Figure 3 shows a plot of uncorrected pairwise sequence identity vs. model corrected evolutionary distances. In these graphs, a linear relationship between both measures (dashed line) suggests that genetic saturation is not an issue (yet). Deviation from this linear suggests increasing genetic saturation [29]. Due to the close relatedness of the types classified in the genus *Alphapapillomavirus* (65.9% mean pairwise sequence identity), nucleotide-based alignments were feasible (Figure 3). However, with more and more diverse papillomaviruses being identified, genetic saturation is a real concern (Figure 3) and thus we need to account for forward and backward substitutions. While the use of evolutionary models can help to alleviate this problem during tree construction [24,34], simply calculating pairwise identities is destined to dramatically underestimate the true sequence divergence, thus skewing the classification of these viruses. In addition, the curves (Figure 3) appear to be asymptotic to about 60% uncorrected distance. This implies that the 60% genus demarcation may be, in part, driven by saturation of the data.

## 7. Genetic Saturation Blurs the Existing Genus Demarcation Criteria

**Figure 4:**
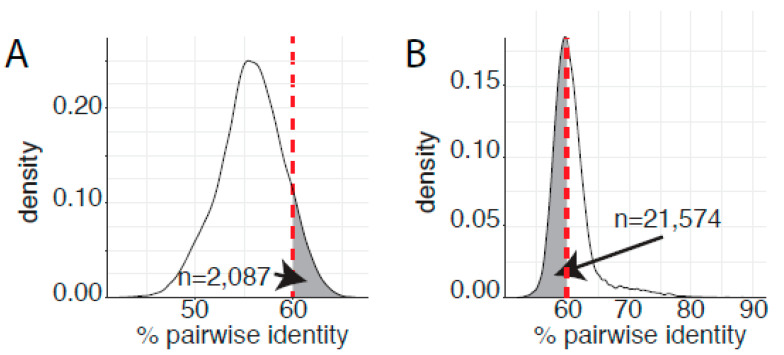
Genetic saturation blurs the demarcation criteria. (**A**) Pairwise L1 sequence identities for viral types classified as belonging either to the *Alphapapillomavirus or Gammapapillomavirus* genera were calculated as in Figure 2 and plotted. The fraction of pairwise comparisons that share more than 60% sequence identity, and should therefore belong to the same genus, is highlighted by grey shading. (**B**) Pairwise L1 sequence identities for viral types classified as belonging to the genus *Gammapapillomavirus* were calculated as in Figure 2 and plotted. The fraction of pairwise comparisons that share less than 60% sequence identity, and should therefore belong to separate genera, is highlighted by grey shading.

Since 2004, papillomavirus classification uses 60% sequence identity as the criteria to assign viruses to distinct genera [24]. A phylogenetic tree of the *Papillomaviridae* clusters most human papillomaviruses into three main clades corresponding to the genera *Alphapapillomavirus*, *Betapapillomavirus*, and *Gammapapillomavirus* (Figure 1). Viruses with the genera *Betapapillomavirus* and *Gammapapillomavirus* are primarily commensal infections of the skin. However, specific viruses within each genus are likely associated with malignant transformation. State-of-the-art molecular evolution analyses demonstrate that both genera diverged ~100 million years ago [4]. Figure 1 shows the phylogenetic position of the genera *Betapapillomavirus* and *Gammapapillomavirus*. Importantly, the viruses in these genera share a most common recent ancestor with non-human viruses in diverse genera *Pipapillomavirus*, *Dyolambdapapillomavirus*, *Dyoetapapillomavirus*, *Treisetapapillomavirus*, *Dyoxipapillomavirus*, and *Taupapillomavirus* (red dot in Figure 1). Considering the evolutionary time passed since these viruses diverged and the association with a wide array of hosts, these viruses should probably belong to separate genera. Therefore, based on the classification criteria, viruses in the genera *Betapapillomavirus* and *Gammapapillomavirus* should not share more than 60% sequence identity across the L1 open reading frame. However, pairwise sequence comparisons between all viruses in either the genera *Betapapillomavirus* and *Gammapapillomavirus* indicate that 2087 sequence pairs share more than 60% sequence identity (Figure 4A). Therefore, if we strictly apply the current classification criteria, all the viruses in these distinct genera should be included in the same genus. Furthermore, when comparing all viruses in the genus *Gammapapillomavirus*, more than 21,000 sequence pairs share less than 60% sequence identity. This would argue that the genus *Gammapapillomavirus* should likely be split into multiple genera (Figure 4B). In summary, the 60% sequence identity demarcation criteria suggest that the genus *Gammapapillomaviridae* should be both split and lumped together. This is non-sustainable and should be addressed to ensure that papillomavirus classification continues to serve the community and facilitates comparative genomics efforts.

## 8. Robust Evolutionary Relationships as the Base for an Updated Taxonomy

While I believe that it is essential to update the current papillomavirus taxonomy, I acknowledge that the existing classification scheme has been highly successful and has been adopted by the papillomavirus community. Therefore, it will be essential to minimize disruptions to the current accepted classification system. 

I believe that a classification system that is formally based on evolutionary histories can achieve both goals of maintaining some of the current accepted genera and species while bringing the taxonomy in line with the current papillomavirus diversity. Furthermore, this would bring the papillomavirus taxonomy into agreement with a recent ICTV consensus (Simmonds et al., 2022 Unpublished).

Several groups have shown that a phylogenetic tree based on three core proteins (E1, E2, and L1) produces a robust reconstruction of the evolutionary history of the *Papillomaviridae*. This phylogenetic tree should be the basis for the taxonomy, ideally using an automated algorithm. To minimize the disruption to the current system, I propose that this depth-first algorithm is trained on the genus *Alphapapillomavirus* as it is currently defined. Practically, the algorithm initially determines the whole-tree distance distribution. Next, starting from a root node the reliability and distance distribution for the *Alphapapilomavirus* clade is calculated. This process is repeated on other subtrees that meet the clustering conditions defined for the *Alphapapilomavirus* clade. Single types, not belonging to a specific genus are termed as *orphan* viruses, until their evolutionary (and taxonomic) position can be reliably confirmed.

The proposed classification scheme uses an E1, E2, and L1 protein based phylogenetic tree to define genera and species. However, viral types will still be defined based on pairwise sequence identity across the L1 ORF. It has been reported that phylogenetic trees based on L1 and E1 are often incongruent [37,38]. This means that it is possible that a virus identified as the closest neighbor based on the E1, E2, L1 phylogenetic tree, is not the closest neighbor based on L1 nucleotide identity. In this case, I propose that a virus would be considered a novel type if it shares less than 90% identity with its closest neighbors in the E1, E2, L1 phylogenetic tree. 

I believe that this proposal to update to the classification scheme will be more robust. Nonetheless, it is paramount that both the initial grouping criteria and downstream demarcation cutoffs are reviewed on a regular basis and updated as needed.

## 9. Closing Remarks

The use of the 2004 taxon demarcation thresholds has resulted in a dramatic increase in the number of species and genera with the family *Papillomaviridae*. Furthermore, many genera and species consist of just a single viral type [1,2,32,39]. Whether the dramatic increase in the number of genera and species is an issue, depends on primarily on one’s philosophical views on lumping and splitting. As George G. Simpson put it, *“splitters make very small units-their critics say that if they can tell two animals apart, they place them in different genera … and if they cannot tell them apart, they place them in different species. … Lumpers make large units-their critics say that if a carnivore is neither a dog nor a bear, they call it a cat.”* [40]. However, the current classification system has shortfalls that need to be addressed. This is specifically the case if species or genus membership is used as the basis for comparative genomic studies or as a basis to extrapolate physiological properties to related viruses.

## Data Availability

Not applicable.
